# A Case of Anomalous Origin of the Right Coronary Artery from the Left Sinus of Valsalva with a Malignant Course

**DOI:** 10.7759/cureus.5794

**Published:** 2019-09-28

**Authors:** Amol Gupta, Vinod Kumar, Ravi Gupta, Samir Samarany

**Affiliations:** 1 Cardiology, Heart, Vascular, and Leg Center, Bakersfield, USA

**Keywords:** congenital heart disease, coronary artery anomaly, right coronary artery, computed tomography, coronary angiogram

## Abstract

Congenital heart disease in adults, including congenital anomalies of the coronary arteries, can be asymptomatic and diagnosed incidentally, but they can also be a cause of sudden cardiac death. The recent guidelines on the management of adults with congenital heart disease from the American Heart Association (AHA) and the American College of Cardiologists (ACC) identify that an anomalous coronary artery origin can lead to myocardial ischemia, arrhythmias, or sudden cardiac death. When the course of the coronary artery runs between the aorta and pulmonary trunk, it is described as having a "malignant course." Emergency surgical correction is required to restore the normal anatomy of the aberrant coronary artery. This report is of a 57-year-old man with a history of hypertension who had a normal electrocardiogram (ECG). A nuclear exercise stress test showed a resting and exercise ejection fraction (EF) of 56% with transient ischemic dilatation (TID) of the left ventricle. Coronary artery computed tomography angiography (CTA) identified an anomalous right coronary artery (AORCA) originating from the left sinus of Valsalva and coursing between the aorta and pulmonary trunk. TID on nuclear imaging is usually associated with left ventricular hypertrophy, microvascular disease, or multivessel macrovascular disease and has not been previously described in AORCA.

## Introduction

Congenital heart disease in adults, including congenital anomalies of the coronary arteries, can be asymptomatic and is diagnosed incidentally. Anatomical variants of the coronary arteries are common and usually have no clinical significance [1]. However, recent guidelines from the American Heart Association (AHA) and the American College of Cardiologists (ACC) on management of adults with congenital heart disease have highlighted that an anomalous coronary artery origin with a subsequent malignant course (i.e., the course of the artery is between the aorta and pulmonary trunk) can lead to myocardial ischemia, arrhythmias, or sudden cardiac death [2-3]. Thus, surgical correction is required to restore the normal anatomy of the aberrant coronary artery with the malignant course [4-5]. The most common coronary artery anomaly is an origin of the left main coronary artery or left anterior descending (LAD) coronary artery from the right coronary sinus [6].

We present a case of a 57-year-old man with an incidental finding of anomalous origin of the right coronary artery (AORCA) from the left sinus of Valsalva; we describe the diagnostic findings and discuss the approach to diagnosis and the lessons that can be learned from this case.

## Case presentation

A 57-year old man, who had recently emigrated to the United States from Syria, was referred by his primary care physician for cardiology review and management of hypertension. He attended the outpatient clinic with his daughter, who acted as his translator. He had noted that he was getting easily tired with usual activity. He also needed to renew his medications, including amlodipine, benazepril, and aspirin. He denied recent symptoms of fever, weight loss, weakness, headache, cough, or chest pain. He had been diagnosed with hypertension eight years previously following the investigation of symptoms of headache.

In his medical history, he had been a smoker for the past 15 years but with no history of alcohol or recreational drug use. He had no previous history of surgery. His mother had a history of hypertension, type 2 diabetes mellitus, and coronary artery disease (CAD) and died from myocardial infarction (MI) at the age of 83 years. His father had died from a stroke, and his younger brother died suddenly at the age of 43 years from an unknown cardiac event.

On physical examination, he was well-nourished with a body mass index (BMI) of 27.47 kg/m2. He had no signs of cyanosis or jaundice. Cardiovascular examination showed normal rhythm, with no cardiac murmur. There was mild bilateral ankle edema. Examination of all other systems was normal. His blood pressure (BP) was 128/78 mmHg, his pulse was rate 67 bpm, and his oxygen (O2) saturation on air was 95%. His electrocardiogram (ECG) findings were normal, and he had normal sinus rhythm. His most recent low-density lipoprotein (LDL) was 138 mg/dL (normal, <100 mg/dL), and triglyceride (TG) was 103 mg/dL (normal, <150 mg/dL).

**Figure 1:**
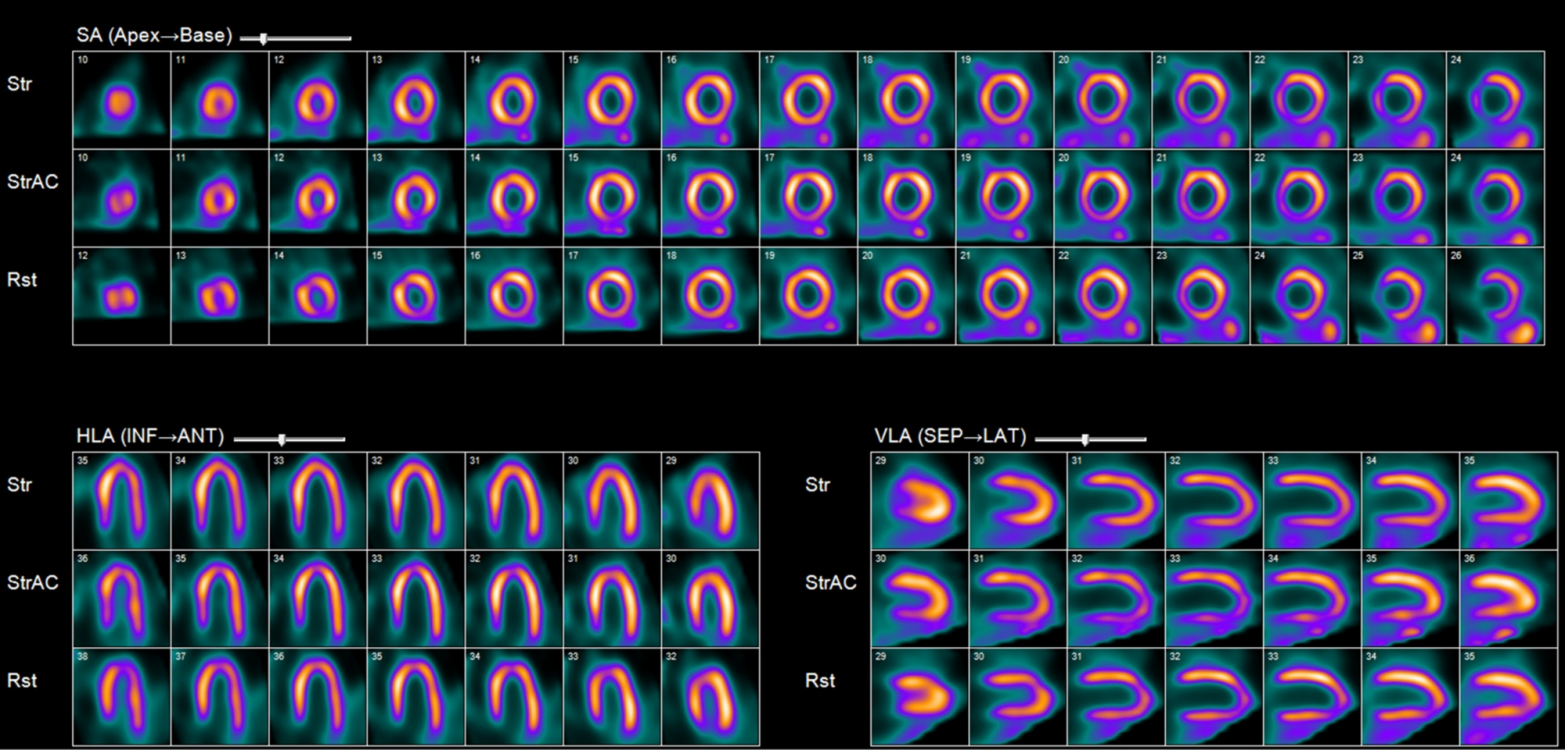
Nuclear stress testing, or stress myocardial perfusion imaging Resting and exercise ejection fraction of 56% with transient ischemic dilatation. ANT=anterior; HLA=horizontal long axis; INF=inferior; Lat=lateral; Rst=rest; SA=short axis; SEP=septum; Str=stress; StrAC=attenuation-corrected stress; VLA=vertical long axis.

Given his clinical presentation and family history of heart disease, his initial suspected diagnosis was of possible CAD. An echocardiogram and nuclear exercise stress test were ordered. His echocardiogram showed a resting ejection fraction (EF) of 70% with an otherwise normal study. A nuclear exercise stress test, or stress myocardial perfusion imaging (MPI), showed a resting and exercise EF of 56%. There was transient ischemic dilatation (TID), which suggested left ventricular hypertrophy or ischemic dilatation due to microvascular or coronary artery disease (Figure 1).

**Figure 2:**
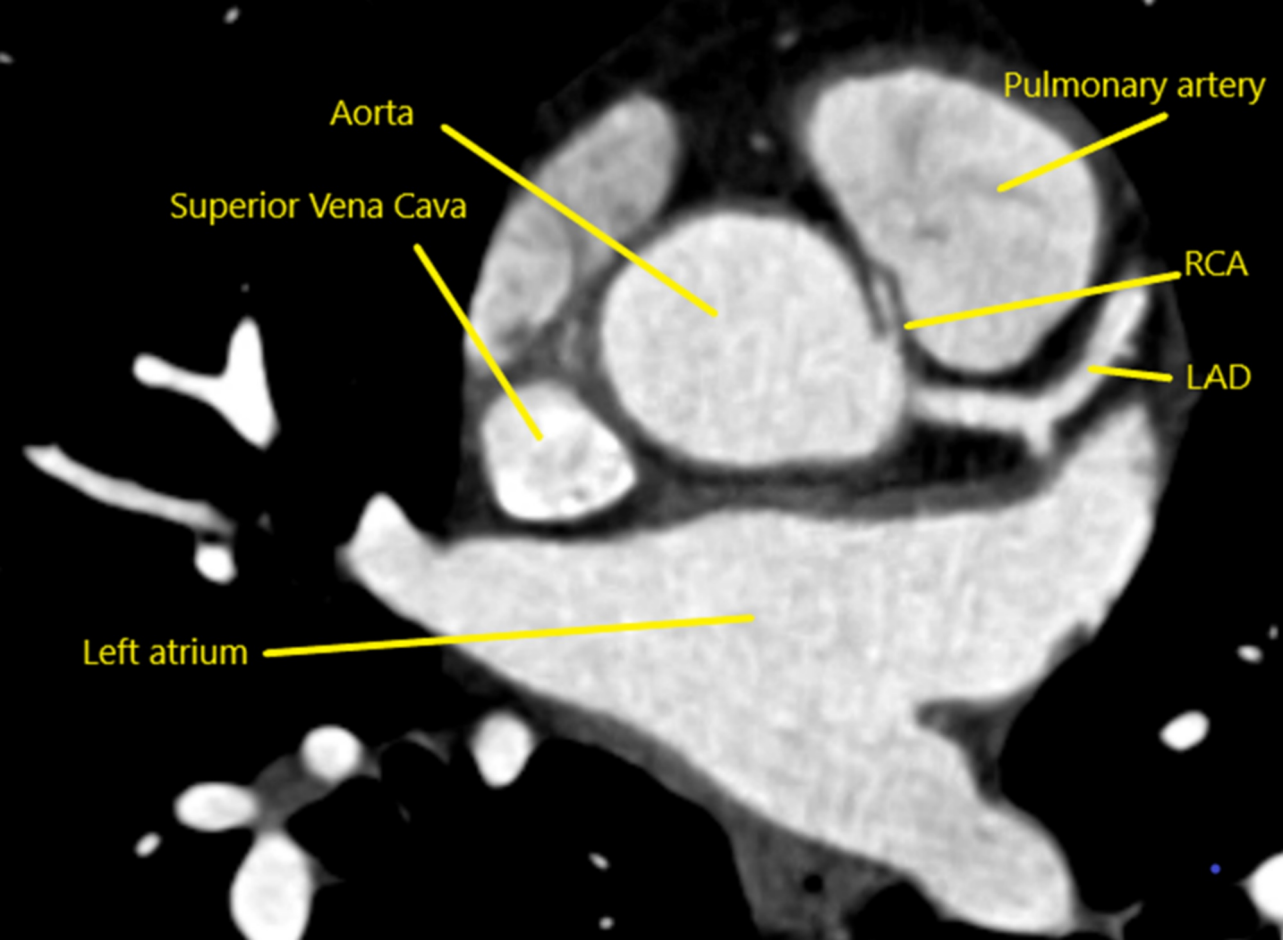
Computed tomography angiography, anomalous origin Anomalous origin of the right coronary artery from the left sinus of Valsalva. LAD=left anterior descending artery; RCA=right coronary artery.

**Figure 3:**
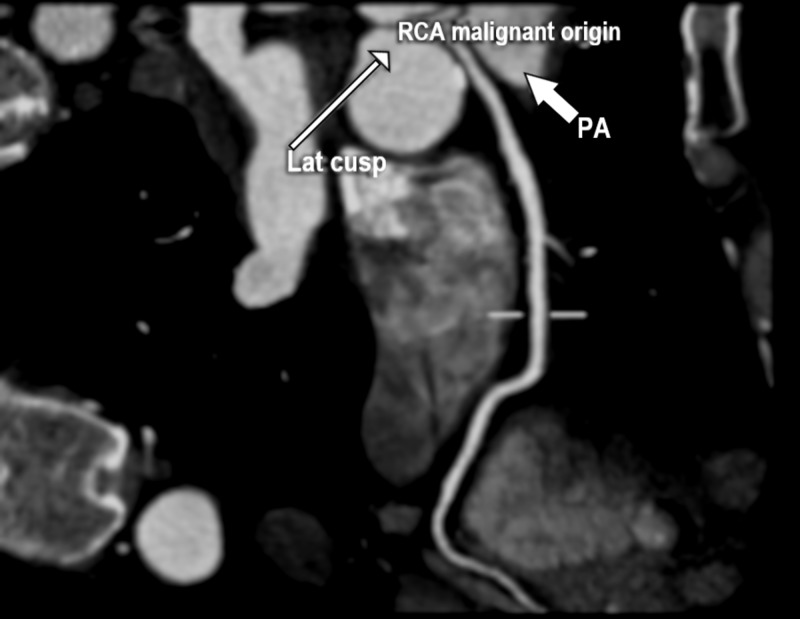
Computed tomography angiography, malignant course Malignant course of the right coronary artery between the aorta and the pulmonary trunk. Lat cusp=lateral cusp of aorta; PA=pulmonary artery.

Two weeks later, computed tomography angiography (CTA) was performed, which showed an anomalous origin of the right coronary artery (AORCA) from the left sinus of Valsalva, with a malignant course between the aorta and the pulmonary trunk (Figures 2-3). Because the aberrant course of the right coronary artery (RCA) made it susceptible to compression during increased cardiac output and could result in sudden cardiac death, he was referred for cardiothoracic surgery for an urgent unroofing procedure to restore the normal anatomy of the RCA. The patient successfully underwent surgery. 

## Discussion

The anomalous aortic origin of a coronary artery occurs rarely and has a reported incidence of 0.64% of live births [7], with the prevalence of a RCA arising from the left sinus of Valsalva being estimated at 0.17% [8-9]. This case has demonstrated that patients with AORCA with a malignant course (i.e., the course of the artery is between the aorta and pulmonary trunk) can be asymptomatic and diagnosed in middle-age. The clinical challenge is that when diagnosed, these cases should be treated as an emergency and corrective surgery should be performed to prevent myocardial ischemia, arrhythmia, or sudden cardiac death due to compression of the aberrant coronary artery between the aorta and the pulmonary artery [10]. AORCA with a malignant course can be associated with angina, exercise-induced syncope, cardiac arrhythmias, myocardial ischemia, or cardiac arrest [10-11]. This patient had symptoms of progressive fatigue, had a history of hypertension and hyperlipidemia and a family history of ischemic heart disease (IHD), which would have increased his risk of CAD and IHD. In his family history, a younger brother had died suddenly from an unknown cardiac cause, which raises the possibility of a familial form of AORCA or other congenital heart diseases [12]. Anomalous origin of the coronary arteries from the aorta can be a component of complex congenital heart conditions that include tetralogy of Fallot, transposition of the great arteries, double-outlet right ventricle, transposition of the great arteries, and truncus arteriosus [2].

This case highlights the importance of further investigations in cases of AORCA in adults, particularly in middle-aged adults with risk factors for IHD, as the results may guide clinical management [5]. This patient had a normal ECG, and although an ECG stress test was not performed, nuclear stress testing, or stress MPI, showed a resting and exercise EF of 56% with TID (Figure 1). Hypertension may cause TID in cases where it causes left-ventricular hypertrophy; however, this patient had a history of well-controlled hypertension and no left-ventricular hypertrophy was observed on ECG and thus further workup for TID was not performed. Although TID has not been previously reported in association with AORCA with a malignant course, in patients undergoing stress MPI, the presence of TID has been shown to specifically detect extensive or severe CAD [13]. In this case, the finding of TID initially suggested left ventricular hypertrophy or ischemic dilatation due to microvascular disease or CAD.

Echocardiography, cardiac magnetic resonance angiography (MRA), and coronary CTA may be used to identify the anomalous origin and course of the RCA. Although CTA uses radiation and requires the use of contrast, it has been shown to have high resolution and can identify the course of the coronary artery in relation to adjacent anatomy and can visualize the coronary ostia [4]. The use of fractional flow reserve CT (FFRCT) has been shown to identify ischemic changes associated with AORCA [14]. In this case, coronary CTA provided the diagnosis of AORCA and, as shown in Figure 2, CTA also provided an accurate anatomic assessment of the course of the anomalous coronary artery.

In this case, the patient was scheduled for cardiac surgery with unroofing, or marsupialization, of the right coronary artery to prevent compression during its malignant course [15]. In all age groups, the management of patients with anomalous origin of the coronary arteries is highly variable, ranging from conservative management to surgical correction, possibly because this condition can present at any age [16-17]. The procedure of unroofing, or marsupialization of the coronary artery is a procedure that can be performed with minimal risk and usually results in good anatomic and functional outcome [15]. The range of treatment approaches highlights the need to establish consensus clinical guidelines for patient management. Recently, a working group from the American Association for Thoracic Surgery (AATS) have developed the first consensus clinical practice guidelines for the surgical management of anomalous aortic origin of the coronary arteries [17].

## Conclusions

We have described the case of a 57-year-old man with an incidental finding of AORCA arising from the left sinus of Valsalva. The approach to diagnosis, in this case, highlights the importance of evaluating not only the anomalous course of the coronary artery but also the associated degree of cardiac ischemia before planning treatment that is individualized for the adult patient with this rare form of congenital coronary artery anomaly.
